# Robotic Near-Total Pancreatectomy for Nesidioblastosis after Bariatric Surgery

**DOI:** 10.1007/s11695-016-2318-6

**Published:** 2016-10-07

**Authors:** Antonio Luiz de Vasconcellos Macedo, Jairo Tabacow Hidal, Wagner Marcondes, Fernando Concilio Mauro

**Affiliations:** Hospital Israelita Albert Einstein, Av. Albert Einstein, 627 - 5° Andar - Salas 512 e 514, Sao Paulo, 05652-900 Brazil

**Keywords:** Pancreatectomy, Nesidioblastosis, Robotic, Bariatric, Obesity

## Abstract

Postprandial symptoms of neuroglycopenia after bariatric surgery may result as a consequence of endogenous hyperinsulinemic hypoglycemia (nesidioblastosis) not dumping syndrome. Pancreatectomy is an acceptable treatment for this condition. We present the video of a case of near-total distal robotic pancreactectomy for the treatment of nesidioblastosis after Roux-en-Y gastric bypass. Robotic pancreatectomy is an alternative to the treatment of nesidioblastosis after Roux-en-Y gastric bypass.

**Electronic supplementary material:**

The online version of this article (doi:10.1007/s11695-016-2318-6) contains supplementary material, which is available to authorized users.

## Introduction

In 2005, Service et al. [1] called attention for the first that postprandial symptoms of neuroglycopenia after gastric bypass may develop as a result of pancreatogenous hyperinsulinemic hypoglycemia not dumping syndrome. Although rare, several other cases have been reported [2]. Pancreatectomy is an acceptable treatment for this condition [1–3]. We aim to present a video of a case of near-total distal robotic pancreactectomy for the treatment of nesidioblastosis after Roux-en-Y gastric bypass.

## Case

A 34-year-old obese female patient underwent a laparoscopic Roux-en-Y gastric bypass (body mass index 41 Kg/m^2^). Episodes of hypoglycemia erroneously attributed to dumping syndrome started 5 months after the operation. The Roux-en-Y gastric bypass was converted to a sleeve gastrectomy (body mass index 23 Kg/m^2^) after 4 years. The new operation was not successful in controlling the episodes of hypoglycemia. A full workup excluded an insulinoma and the diagnosis of nesidioblastosis was made. A robotic subtotal pancreatectomy was performed. Operative time was 7 h. A self-limited pancreatic fistula was the only complication associated to the procedure. No intervention was necessary to treat the fistula. The patient was discharged at postoperative day 5. Pathologic examination of the pancreas confirmed the diagnosis of nesidioblastosis. No episode of hypoglycemia or pancreatic insufficiency was observed with a follow-up of 2 months.

## Discussion

Hypoglycemia is a rare event after restrictive bariatric operations but it has an incidence of 0.2 % after Roux-en-Y gastric bypass [4]. Different surgical procedures have been described to treat postoperative hypoglycemia: gastric tube placement, reversal of the bypass with or without concomitant sleeve resection, gastric pouch restriction, and pancreatic resection and re-resection [2]. Pancreatectomy is not the primary choice; however, if a conversion to a restrictive procedure fails, options are few. Pancreatectomy is a not a simple procedure per se. Pancreatectomy after a bariatric surgery adds the difficulty of the overweight if weight loss was not achieved yet, adhesions from the previous abdominal operation and a Roux-limb crossing over the pancreas. Robotic pancreatectomy is a valuable minimally invasive approach to these problems.

## Electronic supplementary material

ESM 1 (MP4 1.14 GB)
